# The traits of Autism Spectrum Disorder and bullying victimization in an epidemiological population

**DOI:** 10.1007/s00787-023-02228-2

**Published:** 2023-05-23

**Authors:** Maria Junttila, M. Kielinen, K. Jussila, L. Joskitt, M. Mäntymaa, H. Ebeling, M.-L. Mattila

**Affiliations:** 1 Clinic of Child Psychiatry, Faculty of Medicine, University of Oulu, P.O. Box 2000, 90014 Oulu, Finland; 2 Nuorten Ystavat, NGO, Oulu, Finland; 3 Research Unit of Clinical Medicine, Child Psychiatry, University of Oulu, Oulu, Finland; 4 Division of Psychology, VISE, Faculty of Education and Psychology, University of Oulu, Oulu, Finland

**Keywords:** Traits of Autism Spectrum Disorder, Autism, ASD, Victimization, Bullying, ASSQ, Total population

## Abstract

Autistic children (Autism Spectrum Disorder, ASD) show an increased risk of bullying victimization and often face challenges in communication and peer relationships. However, it is unclear to what extent the amount and quality of ASD traits are associated with bullying victimization. This study examined the association of bullying victimization and ASD traits in an epidemiological population of 8-year-old children (n = 4408) using parent and teacher completed Autism Spectrum Screening Questionnaires (ASSQs), both separately and combined. The ASSQ items relating to loneliness and social isolation, lack of co-operating skills, clumsiness and lack of common sense were associated with victimization in the study population. The higher the ASSQ scores, the more the children were victimized: the ASSQ scores increased in parallel with victimization from 0 (0% victimized) to 45 (64% victimized). The victimization rate was 46% in ASD sample, 2% in the total population sample and 2% in the non-ASD population sample. The results enable more targeted means for recognizing potential victimization.

## Introduction

Bullying is commonly defined as repetitive, intentional negative action that causes or attempts to cause fear, discomfort, or injury to another person. It can be physical, verbal, social or psychological. There is an imbalance of power between the bully and the victim [1]. Bullying is a worldwide phenomenon and a major problem in schools: different studies have given prevalences of 10% up to 45% for school-aged children [2, 5–8] albeit there is a significant global variation in the rates, from 8.5% in Europe to over 40% in Eastern Mediterranean areas and Africa [2]. The Finnish National Institute for Health and Welfare publishes an annual School Health Promotion Study [3], and according to that study 7.3% of Finnish 10- to 12-year-old faced bullying in school at least once a week in 2017, reported by their parents. Santalahti et al. [4] have reported victimization rates in a total population sample of 8-year-old in which they used parents, teachers, and children themselves as informants. According to parents, 11% of the children were somewhat bullied and 1% were definitely bullied, according to teachers, 5% were somewhat bullied and less than 1% were definitely bullied. The 8-year-old themselves responded that 23% of them were bullied sometimes and 3% almost every day.

During their school years, children and adolescents go through a unique and momentous period both socially and biologically, and bullying can be considered as a major risk factor for their wellbeing, with far-reaching negative effects on the victim, predisposing to psychiatric symptoms such as depression, panic disorder and anxiety, and even suicidal feelings and behavior in the victim’s early adulthood [9–13]. Positive experiences during the early years are crucially important to children’s ability to lead a healthy life later on, and the World Health Organization (WHO) has named positive experiences in education as a key element to reduce global health inequities [14].

A bullying situation was first recognized by Salmivalli et al. [15] as a group process in which the participants have different roles such as victims, bullies, reinforcers and assistants of the bullies, defenders of the victims, and outsiders. Since then, other studies have also conceptualized bullying as a group phenomenon and as part of the socio-ecological structures that surround a child [16–18]. One crucial structure is peer ecology, which includes the complex structures of behavioral norms and social status hierarchies that are assumed to play a significant role in victimization [19–22]. The bullying acts as an effective quest for power and high status, and those who are unlikely to stand up for themselves are often selected as victims [23–25]. A healthy communication and assertiveness are suggested to be important protective factors against victimization, which is why children with impairments in peer communication are at higher risk of being victimized [25–27]. Systematic reviews provide evidence that whole-school interventions such as the KiVa anti-bullying program are very effective at reducing both bullying and victimization [28].

One of the impairments causing troubled peer-communication is Autism Spectrum Disorder (ASD), a pervasive neuropsychiatric disorder typically manifested by deficits in two main domains: limitations in social communication and interaction, and stereotyped, repetitive patterns of behavior [29]. A global median autism prevalence ranging within and across regions is estimated to be 1% (range 0.01 to 4.36%) [30–32]. However, ASD is now also recognized as a spectrum where autistic traits are continuously distributed across the population and range from very mild to severe [33–36]. ASD creates challenges in the child’s understanding of social communication cues [37] and makes it difficult to form and maintain interpersonal relationships. Autistic children have difficulties in context utilization in comprehension [38, 39] and in staying on topic and providing relevant information in conversation [40]. They also tend to approach others in unconventional ways [41], are poorly equipped for social interaction, struggle particularly in creating and maintaining peer relationships, and are at increased risk of being victimized [42].

There is a growing number of studies published about victimization of autistic children, and their victimization rate has shown to be considerably higher compared to victimization in general population [43–47]. The previous studies regarding bullying victimization in autistic children and youth have used variable evaluators (parent, self, teacher, peer) and measurements (interviews, questions, standardized and un-standardized victimization questionnaires) to assess victimization, and the results have varied from peer rated 7% [48] to parent rated 94% [49]. Several predictors for victimization have also been found: higher internalizing problems and conflicts with peers [50], younger age and lower number of friends [47], household income, race, social- and communication skills and self-concept [74], behaviour problems [51], attention-deficit hyperactivity and depression [52], high levels of ASD traits and inclusive classroom settings [53]. Hebron et al. [54] found a cumulative risk effect on the victimization: exposure to bullying increased as the number of risk factors increased.

Studies evaluating the amount and quality of more specific ASD traits and their effect on victimization are however more scarce: a previous study by Sofronoff et al. [55] found a connection between victimization and social vulnerability (e.g., naivety, trust) in autistic population of 6–16 year-olds (n = 133), while Forrest et al. [42] compared the victimization rate and Children’s Social Behavior Questionnaire (CSBQ) subscales in autistic population and found a connection between victimization and two features: *resistance to change* and *not being optimally tuned to the social situation.* Rai et al. [56] studied depression and ASD / ASD traits (four subcategories: social communication, coherence, repetitive behavior, and sociability) in childhood in general population, and reported strongest association between social communication difficulties and bullying at the age of 10. When these two factors were co-occurring, they importantly contributed to depression later on. Other than their study, to our knowledge, there have been no studies connecting bullying victimization and the specific traits of ASD in a general child population. Therefore, we aimed to see whether certain ASD traits increase the risk of victimization among a total population of 8-year-old children. Since one can now consider ASD as a continuum, our aim was also to determine whether the amount of ASD traits increases victimization. Lastly, we estimated the bullying victimization rate in a total population of 8-year-old children, in a non-ASD sample, and in an ASD sample.

## Methods

### Participants

The participants consisted of a population of 8-year-old school children (n = 4408; 2237 females, 2171 males, mean age 8.3 years, age range 7.8–8.8 years) in the Northern Ostrobothnia Hospital District area in Finland in 2000. The screening phase of our study using the Autism Spectrum Screening Questionnaire (ASSQ) was carried out via schools. All primary schools (329) with 5484 children in the Northern Ostrobothnia Hospital District area were included in the study, and 321 (98%) agreed to participate. Nine schools, including seven special schools, did not have any pupils in the target age population, and eight schools did not return the study material, so the final number of participating schools was 304 (with 5, 242 children) of which 15 were special schools. The non-participating schools did not differ significantly from the participating schools. Of participating non-special schools, 12 had special classes or integrated settings for pupils with special needs. In the total population sample of 4408 children, 28 were diagnosed with ASD according to DSM-IV criteria.

The study population was homogeneous, mainly of Finnish extraction and Finno-Ugric origin. The Finnish public school system reaches the whole age class and offers free, equal, and good quality learning opportunities for all children aged 7–16 years. All socio-economical groups are represented in the public school system, and private schools are almost non-existent, since the law prohibits schools functioning for economic benefit in Finland. Children with intellectual disability are offered a prolonged time in the comprehensive school, starting at the age of 6 and lasting until the age of 17. The comprehensive school has two phases: primary school from 1st to 6th grade with pupils aged 7–12 years, and secondary school from 7 to 9th grade, with pupils aged 13–16 years [57].

### Measures

*The Autism Spectrum Screening Questionnaire* (ASSQ) [60] is a 27-item questionnaire for assessing autistic traits in 6- to 17-year-old children and adolescents with a full-scale intelligence quotient (FSIQ) equal to or more than 50. The ASSQ, originally in Swedish, has been officially translated into Finnish [59] and validated in Finland [61]. The questionnaire is identical for both parents and teachers and can be completed in 10 min. The respondent indicates if the rated child stands out as different from other children of his/her age. The items are rated on a 3-point Likert-type scale (i.e., normal = 0, some abnormality = 1, and definite abnormality = 2) with total scores ranging from 0 to 54 (0–108 when using summed parent-rated and teacher-rated scores) [53], with higher scores indicating more severe levels of social impairment. In the Finnish translation, for 7- to 12-year-old children (FSIQ ≥ 50), the optimal cut-off scores are 30 in clinical settings and 28 in total population screening using summed parent-rated and teacher-rated ASSQ scores [61]. Additionally, a revised and extended version of the ASSQ, the Autism Spectrum Screening Questionnaire-Revised Extended Version (ASSQ-REV), has been developed in Swedish to better capture the female phenotype of ASD [63].

In our study, the Autism Spectrum Screening Questionnaire (ASSQ) item #25 (“Is bullied by other children”) was used as the indicator of bullying victimization, and parents and teachers as raters, both scoring the ASSQ by giving 0–2 points for item #25. Since previous studies [58, 59] have found a low agreement between these two informant groups, we aimed for a more holistic view and combined the scorings of the raters: two points or more equals victimized, i.e., (a) one (1) point from parent(s) AND one (1) point from teacher*,* or (b) two (2) points from parents and/or teacher. This way we were able to identify the children who were considered victimized to some extent (one ASSQ point from item #25) by both informants, or considered *definitely* victimized (i.e., two ASSQ points from item #25) at least by one of the two informants.

The *Autism Diagnostic Interview-Revised* (ADI-R) [64] and the *Autism Observation Schedule* (ADOS) [65]. The ADI-R is a standardized investigator-based, structured parental interview developed to elicit a full range of the information needed when evaluating the diagnostic criteria of ASD. It covers the main symptom areas associated with ASD: reciprocal social interaction, communication, and restricted and stereotyped behavior and interests. The ADOS is a semi-structured observational assessment of social interaction, communication, and play or imaginative use of materials. It comprises four modules based on the verbal level of the subject being evaluated.

The *Wechsler Intelligence Scale for Children, 3*^*rd*^*. ed.* (WISC-III) [66] is an individual cognitive ability test designed for children aged 6–16. It consists of verbal and performance subtests.

### The Developmental and Background Questionnaire

A 14-item parental questionnaire was designed to gather information about the participants. It includes information about gender of the child, parental educational background and marital status, number of siblings in the family and child’s previous daycare, school history and the diseases: epilepsy, minimal brain dysfunction, spatial learning disability, mildly impaired intelligence any other pre-disclosed disease, sensory functions, hyperactivity, and attention deficit.

### Procedure

The study was approved by the Ethics Committee of the Northern Ostrobothnia Hospital District. The school inspector, superintendents of all 43 municipalities and all 329 school principals were informed, and permission was granted by them to carry out the total population study in their schools. Written informed consent was obtained from parents.

The parents of the children (n = 5484) in the target population were asked to complete the ASSQ and the background questionnaire. The parents of 4424 (84%) children gave consent to participate and the ASSQ for 3751 children (85%, 1–25 items missing in 187 cases) were completed by parents. After parental permission, the teachers of 4382 children (99%, no missing items) completed the ASSQ. Two children with consent from parents were not rated by either parents or teacher. Thus, 4422 children with parent-rated and/or teacher-rated ASSQs remained.

A high-/medium-risk sample of children for ASD (n = 125) based on high ASSQ scores (the established Swedish cut-off scores for clinical settings, i.e., teacher-rated scores of ≥ 22 and/or parent-rated scores of ≥ 19) [60] and/or medium ASSQ scores (teacher-rated scores of 17–21 [67] OR teacher-rated scores of 9–16 and parent-rated scores 7–18 (the Receiver Operating Characteristic curves had shown a minimum of 9 points for teacher-rated ASSQ scores and a minimum of 7 points for parent-rated ASSQ scores, with a sensitivity of 95% of ASDs) [60] were invited to take part in diagnostic examinations (see details in [61]), and 110 (88%) participated. The ADI-R and ADOS, module 3, were administered and videotaped in order to obtain detailed information for ASD diagnostics, and the WISC-III tests were performed because the ASSQ has been designed for children with FSIQ equal to or more than 50. School day observations of 24 children were executed and patient records were studied.

Four children showed FSIQ scores below 50 and two children with severe physical disability could not be tested reliably. In addition, eight children were reported in the background questionnaire to have moderate, severe or profound intellectual disability, i.e., FSIQ scores below 50. These 14 children were excluded, leaving 4,408 children (FSIQ ≥ 50) with ASSQ ratings for the total population sample.

Using all gathered data, the diagnoses of ASD according to DSM-IV criteria were defined in 26 children, based on consensus among two researchers (experienced pediatrician and child psychiatrist). Two children with an existing clinical diagnosis of ASD in the hospital registers, did not participate in the diagnostic examinations in our study. These two were, however, included in the ASD study group, and therefore the total number of autistic children was 28.

**Fig. 1:**
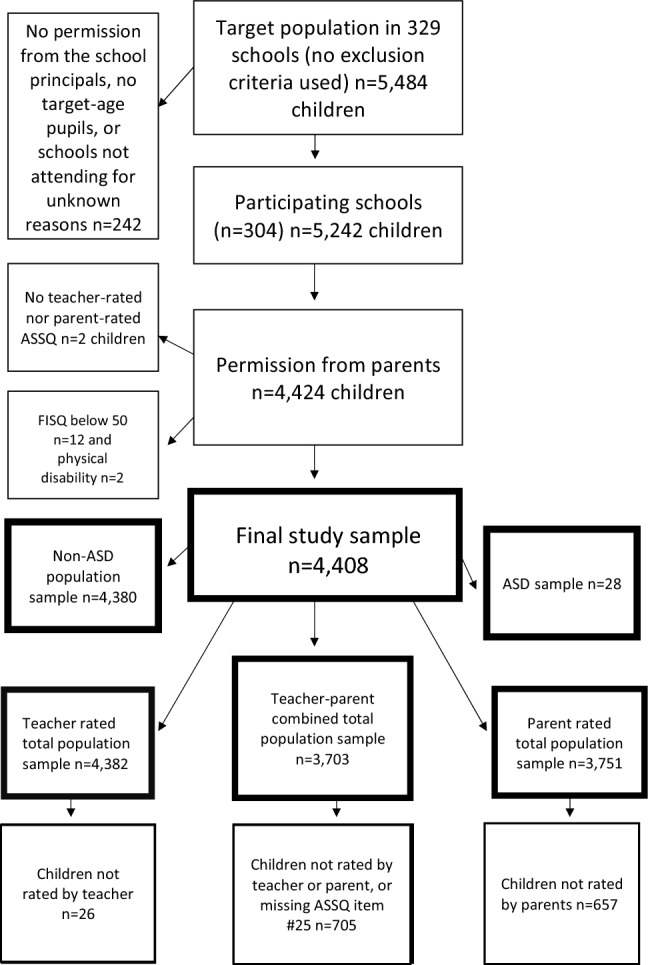
Participant flowchart

The final samples were (1) a total population sample (n = 4408; 80%, 2237 females, 2171 males, mean age 8.3 years, range 7.8–8.8 years), (2) ASD sample (DSM-IV; n = 28, 11 females, 17 males), (3) non-ASD sample (n = 4380), (4) teacher-rated total population sample (n = 4382), (5) dichotomized teacher-parent combined sample (n = 3703) and (6) parent-rated total population sample (n = 3751) as described in the flowchart in Fig. 1.

### Statistical analysis

To assess the prevalence of victimization, the ASSQ item #25 (“is bullied by other children”) was used. We calculated the prevalence of victimization using parents’ and teachers’ assessments separately and used categories (0 = not victimized, 1 = somewhat victimized or 2 = definitely victimized), but in addition we also created a combined parent-teacher informant for the purpose of regression analyses and dichotomized the ratings from parents and teachers as follows: two points or more equals victimized, i.e., one point from parents and one from teacher or two points from parents and/or teacher. In this article, combined teacher and parent ratings are referred to as combined scores and have been used in assessing the overall prevalence of victimization, correlation between each ASSQ item and victimization, and in determining which ASSQ items are mostly associated with victimization.

Statistical analysis was performed with IBM SPSS Statistics v22 software. The Student’s t test was used for continuous variables to determine significant differences between groups. Cross-tabulation was used as a bivariate analysis to find correlation between victimization and each ASSQ items and in evaluating the agreement level of parents and teachers about victimization, and Cohen d and R-squared were used to evaluate the effect sizes in the study groups (parent, teacher and combined parent-teacher). Logistic regression analyses were conducted to explain the relationship between victimization and the sum of ASSQ scores (parent, teacher and combined parent-teacher) adjusted by confounders. Stepwise backwards logistic regression analysis was used to determine the rank of significance within ASSQ items. ASSQ items left in the final model of the analyses were deemed as associated with victimization. Measurements are presented as mean ± standard deviation, percentages and odds ratios (OR) with 95% confidence intervals in logistic regression analysis results. The significance level was set at p < 0.05.

All the ASSQ items that significantly associated at the end of the backward stepwise regression analyses with victimization were adjusted in further separate regression analysis with potential confounders from background questionnaire.

## Results

Parental educational background and marital status, number of siblings in the family and child’s previous daycare history (homecare vs. daycare, from 3-year-old) were not associated with victimization. The following items from the background information form were statistically significantly positively associated with victimization: male gender, epilepsy, minimal brain dysfunction, spatial learning disability, mildly impaired intelligence, any other pre-disclosed disease, hyperactivity, and attention deficit.

The agreement between teacher’s and parent’s results was 10.3% in the cases who received one point and 10.8% in the cases who received two points for ASSQ item #25.

In the total population, the summed ASSQ score from parents’ and teachers’ ratings was 3.61 ± 6.4, median 1.0 (range 0–65). Zero points were recorded in 33% (n = 1,154) and 28 or more points in 1.3% (n = 56). In combined parents’ and teachers’ ratings for item #25, one ASSQ point was given by both raters for the same child in 55 out of 532 cases and two ASSQ points were given for the same child in 4 out of 37 cases.

### The victimization rates

**Fig. 2:**
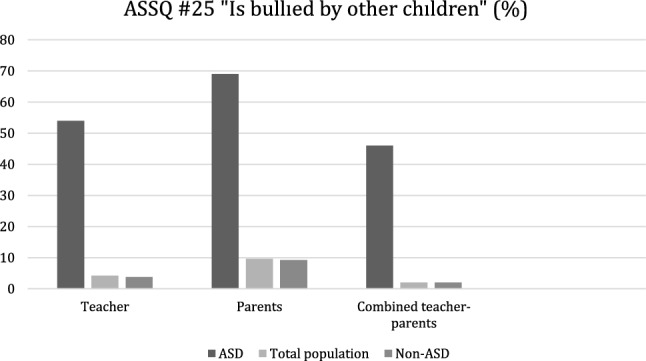
The victimization rates (%) in the ASD sample (n = 28), in the total population sample, (n = 4408) and in the non-ASD sample (n = 4380) as evaluated by the teacher, parent and the combined teacher-parent informant

The combined parents’ and teacher’s ratings showed a victimization rate of 2% in the total population, 46% in the autistic population and 2% in the non-autistic population. The separate parent’s ratings showed victimization rate of 9.6% in the total population, 69% in the ASD population and 9.25% in the non-autistic population. The separate teacher’s ratings showed victimization rate of 4.2% in total population, 54% in autistic population and 3.8% in non-autistic population. The results are presented in Fig. 2.

Separate parents’ results indicated “somewhat” victimized (one ASSQ point) in 9.3% in total population, 54% in autistic population and 9% in non-autistic population and “definitely” victimized (two ASSQ points) in 0.3% in total population, 15% in autistic population and 0.25% in non-autistic population. The separate teachers’ ratings showed “somewhat” victimized (one ASSQ point) in 3.8% in total population, 35% in autistic population and 3.5% in non-autistic population, and “definitely” victimized (two ASSQ points) in 0.4% in total population, 19% in autistic population and 0.3% in non-autistic population.

### The association of the ASSQ scores with victimization

Victimized children had significantly higher summed ASSQ scores than the non-victimized children (23.8 ± 14.1 versus 3.12 ± 5.19, p < 0.001, respectively, Cohen d = 3.719) in the combined parent-teacher scores. When analyzed separately, parent-rated ASSQ scores were 10.1 ± 7.4 (victimized) vs. 1.80 ± 3.08 (non-victimized), p < 0.001, Cohen d = 2.221 and teacher-rated ASSQ scores 13.4 ± 11.1 (victimized) vs. 1.31 ± 3.31 (non-victimized), p < 0.001, Cohen d = 3.027.

Association between victimization and the sum of ASSQ scores without item #25 were not very strong when adjusted with eight confounders (crude ORs were equal to adjusted ORs), parent-rated sum of scores on victimization was 1.2 (95% CI 1.2–1.3), teacher-rated 1.2 (95% CI 1.2–1.2) and combined 1.2 (95% CI 1.1–1.2). The total ASSQ scores without item #25 explains the variance in victimization about 20–30% (parent reported R squared was 0.207, teacher reported 0.263 and combined parent- teacher 0.310).

**Fig. 3:**
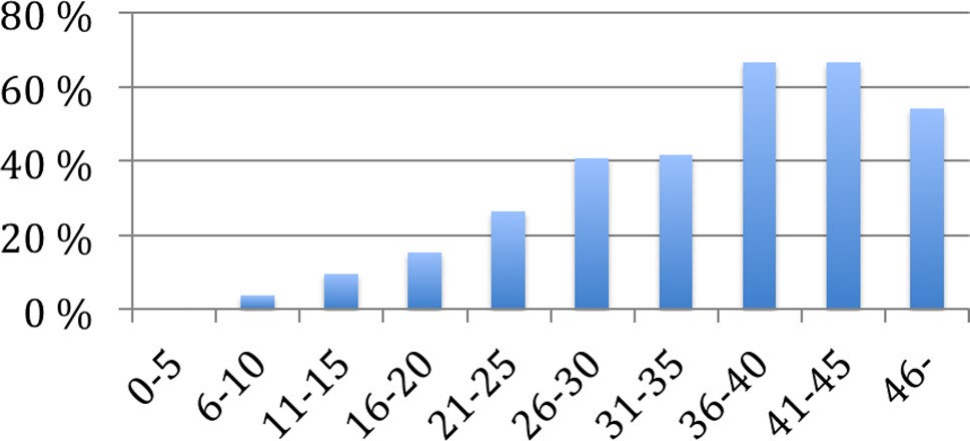
Association of victimization percent and summed parent and teacher ASSQ score in total population sample of 8-year-olds (n = 4408)

The victimization rate increased as the summed parent-rated and teacher-rated ASSQ scores (ASSQ item #25 excluded) increased from 0 (0% victimized,) to 45 points (64% victimized). Eleven children scored higher than 45 and six of them were victimized. The results are illustrated in Fig. 3.

### ASD traits and victimization

The bivariate analysis of each ASSQ item and victimization showed a statistical significance with all ASSQ items in all groups (combined parent-teacher, parents, and teachers). Results are shown in Table 4 in appendix. When analyzed with the backward step-vise regression analysis, there were seven ASSQ items in combined parents’ and teachers’ ratings that were statistically significantly associated with being victimized. The results are shown in Table 1. In separate parents’ ratings, six ASSQ items (Table 2) and in separate teachers’ ratings, four ASSQ items (Table 3) associated with being victimized. One item, #15 (“wishes to be social but fails to make relationships with peers”) was present in all three ratings, both combined and separate. The correlations remained statistically significant after adjusting for confounders (gender, minimal brain dysfunction, epilepsy, spatial leaning disability, mild intellectual disability, any other pre-disclosed decease, hyperactivity, and attention deficit.)

**Table 1:** ASD traits most associated with victimization by combined parents and teacher ratings in the total population sample (n = 3,703)

ASSQ item	OR	95% CI	p value
ASSQ 1 “is old-fashioned and precocious”	4.76	2.73–8.31	< 0.001
ASSQ 13 “makes naive and embarrassing remarks”	11.86	6.28–22.43	< 0.001
ASSQ 15 “wishes to be social but fails to make relationships with peers”	27.42	14.43–52.10	< 0.001
ASSQ 17 “lacks best friend”	13.01	7.90–21.43	< 0.001
ASSQ 18 “lacks common sense”	22.16	11.12–44.16	< 0.001
ASSQ 19 “is poor at games: no idea of cooperating in a team, scores “own goals”	17.02	9.78–29.62	< 0.001
ASSQ 21 “has involuntary face or body movements”	12.31	5.45–27.78	< 0.001

All presented items were separately analyzed with regression analysis adjusted with confounders (gender, epilepsy, spatial learning disability, mildly impaired intelligent, any other predisclosed disease, hyperactivity, and attention deficit)

**Table 2:** ASD traits most associated with victimization by parent ratings in the total population sample (n = 3,751)

ASSQ item	OR	95% CI	p value
ASSQ 1 “is old-fashioned and precocious”	2.26	1.48–3.45	< 0.001
ASSQ 11 “uses language freely but fails to make adjustments to fit social contexts or the needs of different listeners.”	4.91	3.15–7.64	< 0.001
ASSQ 15 “wishes to be social but fails to make relationships with peers”	6.12	3.84–9.75	< 0.001
ASSQ 18 “lacks common sense”	4.95	3.11–7.89	< 0.001
ASSQ 19 “is poor at games: no idea of cooperating in a team, scores “own goals”	4.64	3.20–6.73	< 0.001
ASSQ 20 “has clumsy, ill coordinated, ungainly, awkward movements or gestures”	6.05	3.28–11.16	< 0.001

All presented items were separately analyzed with regression analysis adjusted with confounders (gender, epilepsy, spatial leaning disability, mildly impaired intelligent, any other predisclosed disease, hyperactivity, and attention deficit)

**Table 3:** ASD traits most associated with victimization by teacher ratings in the total population sample (n = 4,382)

ASSQ item	OR	95% CI	P value
ASSQ 12 “lacks empathy”	6.71	4.57–9.86	< 0.001
ASSQ 15 “wishes to be social but fails to make relationships with peers”	9.65	6.48–14.35	< 0.001
ASSQ 17 “lacks best friend”	7.74	5.51–10.87	< 0.001
ASSQ 20 “has clumsy, ill coordinated, ungainly, awkward movements or gestures”	6.53	4.57–9.32	< 0.001

All presented items were separately analyzed with regression analysis adjusted with confounders (gender, epilepsy, spatial leaning disability, mildly impaired intelligent, any other predisclosed disease, hyperactivity, and attention deficit)

## Discussion

The present study gives important insight into the single ASD traits that increase the risk of victimization in the child population. We found five specific traits that were associated with victimization being related to loneliness and social isolation (ASSQ items #15 and #17), lack of co-operating skills (ASSQ item #19), clumsiness (ASSQ item #20), and lack of common sense (ASSQ item #18). The new aspect and strength of our study is the holistic view on victimization that was possible by investigating the combined parent and teacher ratings. Mattila et al. [59] have studied the use of the ASSQ in our epidemiological data and found a slight negative correlation between parents’ and teachers’ ratings in the sample of high-scoring children. They outlined that the use of both informants gives the best understanding. Posserud et al. [36] performed a concordant study in Norwegian total population of 7- to 9-year-old and their finding was, in turn, that parent’s ratings were higher than teachers’, and teachers’ and parents’ agreement on their ASSQ scorings was low to moderate. Thus, in order to prevent a fragmented perception of the phenomenon it is useful to examine different raters’ views on victimization. In addition, the previous studies about ASD victimization have resulted in higher victimization rates by parents [47, 49, 68, 69] compared with teachers [48, 70]. Based on these results we decided to prefer the use of combined answers by teachers and parents when evaluating bullying victimization, but we have also published the separate answers from the informants, since we want to show the differences between the two informants and that way add data for the field of victimization research.

### Social isolation and loneliness

The ASSQ items #15 (“wishes to be social but fails to make relationships with peers”) reflects challenges in forming and maintaining peer relationships that are typical in autism and may lead to social isolation and loneliness, that has a high prevalence in autism [71]. This ASSQ item was yielded in all three analyses (combined parent and teacher ratings, separate parent ratings, and separate teacher ratings). This finding highlights the social nature of victimization phenomena in which social relationships with peers are a key element in establishing a place in the social hierarchy where one can avoid bullying victimization. The above result is in accordance with a recent study of Matthias et al. [72] that found an association between social and communication skills and victimization in ASD population aged 11–22. Based on our data, children as young as eight years distinguish each other by the level of social and communication skills, and a lack of them can lead to bullying victimization. At this age, the children’s circles of friends are possibly not yet fully established, and other children do not easily accept a child whose social communication is different. Rai et al. [56] conducted a population-based study in child population of 10-year-old and discovered an association between ASD/high amount of ASD traits, social communication impairments and bullying victimization. In their study they assessed four core symptoms of ASD (social communication, coherence, repetitive behavior, and sociability) of which bullying was mostly associated with social communication impairments. Matthias et al. [72] also reported the finding that students who were not able to speak clearly were seven times more likely to be victimized. ASD traits commonly include deviant voice pitch, prosody, use of words (“professor-like language”), as well as deviant facial expressions and gaze, and all these non-verbal body language deficits can play a role in failed social communication. Loneliness in autism has been linked to for example sensory avoidance, negative experiences, learned helplessness, anxiety, depression, and camouflaging of autistic traits [73, 74].

Loneliness can cause anxiety that has shown to lead to victimization, and vice versa: victimization causes internalizing problems such as anxiety [8, 75]. According to Jobe and White [76], individuals with a higher amount of ASD traits experience more loneliness, not because of their willingness to be alone, but rather due to their limited social skills. Like a vicious circle, lack of friends prohibits the possibilities to practice social skills, and with limited social skills it is challenging to get close friends. Cresswell et al. [77] also found that autistic adolescents were often lonely even though they would have liked to have friends, and this was rooted in the fact that they did not understand the social settings. Social anxiety and rejection have also predicted victimization [78].

Item #17 (“lack of best friend”) was also associated with victimization in this sample. This is not surprising, since a best friend can function as a shield by standing up to the bully and defending the victim. A lonely and socially isolated child can seem like an easy target. In addition to loneliness, this item may also reflect a preference to spend time by oneself rather than with peers, which is typical in autism.

### Lack of co-operation skills

The ASSQ item 19 (“is poor at games; no idea of cooperating in a team, “scores own goals”) was also associated with victimization and a group setting. Social interaction difficulties increase the risk of withdrawal from situations that demand interplay with other people. In social studies, being sporty has been shown to be associated with a higher prestige in the school hierarchy, especially among boys [79]. In addition, young children tend to be very aware and strict about rules in games and strongly dislike when somebody does not act by them. Classic work by Piaget [80] has recognized the moral developmental phase of 5- to 9-year-old and called it Heteronomous Morality. In this phase, rules are considered to be absolute, and they cannot be changed. At this age, children are still not able to consider, for example, changing the rules even if everybody agrees. Later, Heteronomous Morality is placed by Autonomous Morality, a more relative view on morals. However, there is an insistence on sameness in ASD, and children with ASD traits struggle with change. If the morals change according to the situation, it may cause deep uncertainty in a child with ASD traits, and yet again, make him/her stick out of the peer group. Sometimes co-operation consists of playing according to the rules, but sometimes the best way to co-operate is to be flexible with the rules, and this may be difficult for an autistic child.

### Clumsiness

The ASSQ item 20 (“has clumsy, ill coordinated, ungainly, awkward movements or gestures”) was associated with victimization in both parent-rated and teacher-rated ASSQ, i.e., having easily visible abnormal ways to move oneself is associated with victimization. Alongside with ASSQ item #21 (“has involuntary face or body movements”), these two items were the only features that refer to a physical abnormality and caused victimization. Bejerot et al. [81] have reported that poor gross motor skills are associated with an increased risk of being bullied. In turn, items #26 (“markedly unusual facial expressions”) and #27 (“markedly unusual posture”) were not significant factors in victimization in our study. It seems that mobile physical vs. static appearances were perceived differently, with the former causing victimization. Furthermore, motor skills relate to social skills, since social interaction requires accurate and fine-tuned motor responses as well as good sensorimotor integration: poor timing, poorly integrated movements and responses may lead to barely notable deviations in gestures, facial expressions, prosody etc. that may be considered “strange” and lead to victimization and peer rejection.

### Lack of common sense

The lack of common sense (ASSQ item #18) is a way to indicate behavior that differs from the norm. These norms are often unwritten and unspoken rules that children learn intuitively by watching and listening. Acting against these norms and rules is easily noticed. The definition of “common sense” according to the Cambridge Dictionary is” the basic level of practical knowledge and judgment that we all need to help us live in a reasonable and safe way” [82]. It is considered a positive feature. It is the capability to act rationally in a new context, a way to cut straight to the point and find a simple yet working solution to a problem. It is also knowledge of how things work. A child without common sense may be incapable of solving everyday problems and learning how things work, may ignore unwritten rules and act in a way that is considered peculiar. Especially those who were previously termed subjects with Asperger syndrome may be conspicuously lacking in what the surrounding people consider as common sense [83].

A similar study in a large autistic population would be needed to extract the significant traits causing bullying victimization. Our ASD sample was only 28 children and could not be used in that purpose. One previous study [55] found a connection between victimization and social vulnerability (e.g., naivety) in an autistic population of 6- to 16-year-old in a research sample (n = 133). In our study, naivety (ASSQ item #13; “Makes naïve and embarrassing remarks”) arose as a risk factor for victimization in the total population sample (combined parent and teacher ratings). Forrest et al. [42] have also studied specific ASD symptoms and victimization in an autistic population. They compared victimization rates and the subscales of the Children’s Social Behavior Questionnaire (CSBQ) and found a connection between victimization and two traits: resistance to change and not being optimally tuned to the social situation.

As suspected, according to our study, autistic children end up as targets of peer victimization significantly more often than non-autistic children, and the prevalence of victimization increases as the ASSQ score increases. This is the case from as early on as primary school age. The victimization rate increased to 45 points (64% victimized), after it turned downwards possibly due to small number of bullied children in the high scoring group: only eleven children scored higher than 45 summed ASSQ points in the data, and six of them were victimized. Thus, the number of children with the ASSQ score of 46 or more (n = 11) and the number of victimized children among them (n = 6) were very low, and therefore the victimization rate turning downwards among them may be a chance. In turn, it might also be possible that children with very high ASSQ score are not visibly being bullied, and rather left her/his own devices, but this would need to be a subject for further studies. Almost half of the autistic children were reported as targets of peer victimization by both their parents AND their teachers (46%). In previous studies based on parental report, the prevalence of ASD victimization has varied from 46 to 77% [47, 55, 68, 69, 84]. Our results are in line with this as the prevalence by parental report was 69% in our ASD sample. However, as far as we know, teachers have been used as raters in only two previous studies, and the results have varied from 12% [70] to 30% [48]. In our ASD sample, the prevalence by teacher report was higher, 54%.

It should be stated that there is a lack of reliable, validated instruments for recognizing victimization, and only quite recently there has been more effort to create better instruments, such as for example Child Adolescent Bullying Scale (CABS) [85]. Two of the most widely used longer-standing tools are the Revised Olweus Bully/Victim Questionnaire [86, 87] and the California Bullying Victimization Scale [86], but the bullying victimization studies and surveys have also been using a wide range of different tools to estimate the victimization, often created by researchers themselves. Our approach was to use ASSQ item #25 as a rough indicator for bullying victimization. Because the ASSQ is not primarily a bullying victimization questionnaire it lacks more detailed aspects of bullying, such as description of bullying victimization, timeline for it, different types of bullying (physical, verbal, social, cyber) and there is no self-assessment possibility, as the questionnaire is filled in by the parent(s) and the teacher. The ASSQ offers, however, three options for evaluating current situation in victimization (“is bullied by other children”): “No”, “Somewhat” and “Yes”, which gives an idea of the situation, functions as a quick screening, and opens the door for more detailed victimization analyzes if necessary. Thus, our goal was specially to study the association between bullying victimization and ASD traits by using the ASSQ item #25 as a rough indicator for bullying victimization.

International studies estimate that 10–45% of school-aged children are victimized [2, 5–8]. In our study, the prevalence of peer victimization was 2% among the total child population according to combined parental-teacher report, varying from 4.2% reported separately by teachers to 9.6% reported separately by parents. The results show a low disagreement between parents and teachers as informants, i.e., agreement was 10.3% in the cases who received one point and 10.8% in the cases who received two points for ASSQ item #25. Compared with the School Health Promotion Study by Finnish National Institute for Health and Welfare, the overall victimization rate in our study was considerably lower (7.3% vs. 2%, respectively) The difference, however, could be due to the nature of the ASSQ: the informants may have acknowledged that the questionnaire is for screening autistic features and considered only a certain type of peer bullying as relevant. Yet, Santalahti et al. [4] have reported victimization rates parallel to our data in a total population sample of 8-year-olds.

In our most recent article [88], we extracted factors from the ASSQ in a factor analysis, and interestingly four of the five ASSQ items that were most associated with victimization (#15, #17, #18 and #19) are included in the same factor in our factor model; i.e., these four items are highly interrelated. All these four items are related to social skills, group skills and being able to function as an acceptable part of a group by understanding what is expected from each member. Thus, there seems to be a strong association between victimization and social vulnerability, i.e., between lacking social skills and therefore to be pushed away from others. Sometimes withdrawing is voluntary, but as discussed above, many times children with ASD features lack the skills to be able to form enduring peer relations, and not the will to do so. Our study shows that ASSQ can screen out children with such social challenges. Our study also raises a question of to what extent teachers would benefit from a short question tool to screen for possible victimization, and whether the tool could include ASD traits that most often cause victimization. Extracting the relevant items from the ASSQ and assessing these ASD traits at the end of Finnish preschool, i.e., at the age of seven, might perhaps provide a preventive tool in the battle against victimization in school as the children with social challenges could be quickly identified and start practicing social skills more intensively. There is also evidence that by teaching autistic children not only social skills, but also skills of theory of mind, the victimization decreases [89].

In the present study, we found five relevant features in the ASSQ (items 15, 17, 18, 19, and 20, related to loneliness, lack of co-operating skills, clumsiness, and lack of common sense) that were significantly associated with victimization in both parents’ and teachers’ ratings (either combined or separately). Furthermore, a low outcome score in the ASSQ, i.e., 0–1 points, was strongly associated with lack of victimization. Since bullying victimization is so harmful for all participants, all actions that contribute to decreasing it are very welcome. Teachers have an important role in reducing bullying among autistic pupils. If a teacher does not recognize ASD traits, he or she will often not recognize bullying and may also unknowingly bully a pupil by misinterpreting different school situations. The misinterpretation may occur when the pupil blurts out things or does not follow the teacher’s instructions. In this case, more attention should be paid to in-service teacher training to reduce school bullying among autistic children.

## Conclusions

The ASSQ traits were strongly associated with victimization in both autistic population and in general population of 8-year-olds. Certain ASSQ items, and thus certain ASD traits, were identified as risk factors for bullying victimization in the general child population, while the absence of ASSQ points was an indicator of not being bullied by peers. Efforts to prevent bullying should be made not only with autistic children, but also with general population children who possess one or more of the specific ASD traits causing bullying victimization.

### Limitations

We recognize that using ASSQ item #25 as the indicator for bullying victimization does not include a definition of bullying, neither a timeframe for the bullying. The absence of exact definitions is however a usual feature in autism screening instruments, and they always include interpretations from the informants, in contrast with ASD diagnostic tools which more often offer more specific time frames. Especially when referring to bullying victimization, the informants can have varying conceptions of bullying victimization, and that is why we have formed a combined teacher-parent informant category; to get a more holistic view of the informants. Victimization is a subjective matter and can be interpreted individually even when a definition is used. As a timeframe for victimization in our study, the ASSQ item #25 wording “Is bullied by peers” is in present tense, and therefore refers to the current situation. We also recognize that the embedded question about bullying in the ASSQ questionnaire (item #25) may be a limitation and only give a limited prevalence for victimization, as it did for the overall victimization prevalence, which in our study stayed low compared with the national estimates. However, our more specific results did not diverge from, but were in line with a Finnish survey conducted by Santalahti [4] in an age-group similar to ours and would therefore suggest that the use of ASSQ can be justified at least in an epidemiological sample. In the future, a study with more specific validated victimization tool with more detailed approach to victimization together with the ASSQ could be conducted.

We also notify the limitations regarding the small size of ASD population. Our epidemiological study was conducted in a general population, and even though the sample size was relatively high (n = 4408), the amount of autistic population only included 28 children, since the prevalence of ASD is overall low.

## Data Availability

The data that support the findings of this study are available from the corresponding author, [MJ], upon reasonable request.

## Appendix

See Table 4.

**Table 4:** Group differences of victimized child samples based on scores (1 or more) in each ASSQ item

Shows traits of ASSQ item	Victimized (parent-informed, N = 372)				Victimized (teachers-informed, N = 196)				Victimized (combined parent-teacher-informed, N =87)			
	N (%)	OR	CI (95%)	p	N (%)	OR	CI (95%)	p	N (%)	OR	CI (95%)	p
#1	145 (39%)	2.3	1.9–2.9	< 0.001	77 (39%)	3.8	2.8–5.2	< 0.001	50 (57%)	2.8	1.8–4.3	< 0.001
#2	20 (5%)	2.8	1.7–4.6	< 0.001	25 (13%)	6.8	4.3–10.9	< 0.001	20 (13%)	7.0	4.2–11.9	< 0.001
#3	76 (20%)	6.2	4.6–8.4	< 0.001	92 (47%)	13.5	9.9–18.4	< 0.001	52 (60%)	12.1	7.8–11.9	< 0.001
#4	57 (15%)	3.1	2.2–4.2	< 0.001	38 (19%)	10.2	8.8–15.4	< 0.001	38 (44%)	8.6	5.6–13.5	< 0.001
#5	82 (22%)	4.9	3.67–6.5	< 0.001	41 (21%)	8.8	5.9–13.0	< 0.001	38 (44%)	7.4	4.8–11.4	< 0.001
#6	29 (8%)	4.7	3.0–7.5	< 0.001	44 (22%)	10.0	6.8–14.6	< 0.001	32 (37%)	11.0	7.0–17.5	< 0.001
#7	123 (23%)	3.5	2.8–4.5	< 0.001	48 (24%)	11.4	7.8–16.5	< 0.001	46 (53%)	5.9	3.8–9.0	< 0.001
#8	61 (16%)	5.6	4.0–7.8	< 0.001	82 (42%)	9.4	7.2–13.4	< 0.001	48 (55%)	9.7	6.3–15.0	< 0.001
#9	64 (17%)	4.6	3.4–6.3	< 0.001	44 (22%)	8.8	6.0–12.8	< 0.001	38 (44%)	8.7	5.6–13.5	< 0.001
#10	142 (38%)	6.6	5.2–8.4	< 0.001	49 (25%)	11.1	7.7–16.1	< 0.001	53 (61%)	10.5	6.9–16.3	< 0.001
#11	129 (35%)	7.5	5.8–9.6	< 0.001	69 (35%)	14.6	10.5–20.4	< 0.001	60 (70%)	17.6	11.0–28.2	< 0.001
#12	55 (15%)	5.0	3.5–7.0	< 0.001	78 (40%)	12.8	9.3–17.6	< 0.001	49 (56%)	13.0	8.4–20.1	< 0.001
#13	125 (34%)	6.0	4.6–7.6	< 0.001	66 (34%)	9.7	7.0–13.4	< 0.001	54 (63%)	9.9	6.3–15.5	< 0.001
#14	18 (5%)	5.3	2.9–9.5	< 0.001	57 (29%)	15.2	10.6–21.8	< 0.001	34 (39%)	14.8	9.3–23.4	< 0.001
#15	77 (21%)	7.5	5.5–10.3	< 0.001	97 (49%)	37.0	26.4–51.9	< 0.001	56 (66%)	24.6	15.5–39.3	< 0.001
#16	117 (31%)	7.5	5.7–9.7	< 0.001	91 (46%)	21.8	15.8–30.1	< 0.001	54 (62%)	13.0	8.4–20.4	< 0.001
#17	162 (44%)	5.7	4.5–7.2	< 0.001	131 (67%)	18.7	13.6–22.6	< 0.001	73 (84%)	18.5	10.4–32.9	< 0.001
#18	87 (23%)	6.4	4.8–8.5	< 0.001	64 (33%)	15.6	11.0–22.1	< 0.001	52 (60%)	15.3	9.8–23.9	< 0.001
#19	135 (36%)	7.6	5.9–9.7	< 0.001	90 (46%)	16.3	11.9–22.3	< 0.001	62 (71%)	16.7	10.4–26.9	< 0.001
#20	55 (15%)	10.0	6.8–14.8	< 0.001	77 (40%)	11.7	8.5–16.0	< 0.001	57 (66%)	23.8	15.1–37.7	< 0.001
#21	34 (9%)	5.7	3.7–8.8	< 0.001	37 (19%)	12.4	8.1–19.0	< 0.001	34 (39%)	16.5	10.4–26.3	< 0.001
#22	34 (9%)	7.4	4.7–11.7	< 0.001	37 (19%)	14.3	9.3–22.0	< 0.001	27 (31%)	12.5	7.7–20.3	< 0.001
#23	74 (20%)	4.7	3.4–6.3	< 0.001	36 (18%)	18.6	11.8–29.3	< 0.001	34 (39%)	8.2	5.2–12.8	< 0.001
#24	69 (18%)	5.8	4.3–8.0	< 0.001	33 (17%)	25.5	15.3–42.3	< 0.001	35 (40%)	11.6	7.4–18.2	< 0.001
#26	16 (4%)	9.4	4.6–18.9	< 0.001	32 (16%)	17.2	10.7–27.6	< 0.001	23 (26%)	17.0	10.0–28.8	< 0.001
#27	14 (4%)	32.8	10.7–100	< 0.001	21 (11%)	20.0	11.0–36.4	< 0.001	19 (22%)	25.6	14.1–46.6	< 0.001

Values presented as N (%) of victimized subjects with each ASSQ item

*OR* odds ratio, *CI(95%)* 95% confidence interval
